# Risk Factors of Total Blood Loss and Hidden Blood Loss in Patients with Adolescent Idiopathic Scoliosis: A Retrospective Study

**DOI:** 10.1155/2022/9305190

**Published:** 2022-05-27

**Authors:** Xiangyu Li, Wenyuan Ding, Ruoyu Zhao, Sidong Yang

**Affiliations:** ^1^Department of Spine Surgery, The Third Hospital of Hebei Medical University, 139 Ziqiang Road, Shijiazhuang 050051, China; ^2^Hebei Joint International Research Center for Spinal Diseases, 139 Ziqiang Road, Shijiazhuang 050051, China

## Abstract

**Objectives:**

To investigate the risk factors of total blood loss (TBL) and hidden blood loss (HBL) in adolescent idiopathic scoliosis (AIS) patients undergoing posterior orthopedic surgery.

**Methods:**

The AIS patients who visited department of spine surgery between January 2015 and December 2020 were retrospectively reviewed. Those with a history of posterior orthopedic surgery for AIS were identified, and their clinical data were collected. Gross formula was used to calculate the TBL and HBL. SPSS 20.0 was used for statistical analysis. The potential risk factors of TBL and HBL were assessed by independent *t*-test or univariate analysis. The risk factors of TBL and HBL were determined by multiple linear regression.

**Results:**

A total of 114 patients were included in this study. Operative time (*P* < 0.001), postoperative platelets (PLT) (*P* = 0.001), the number of surgical fixation segments (*P* < 0.001), implanted screws (*P* < 0.001), hospital stay (*P* = 0.006), type of scoliosis (*P* < 0.001), and correction angle of scoliosis (*P* = 0.063) were the potential risk factors of TBL. Operative time (*P* < 0.000), postoperative PLT (*P* = 0.095), the number of surgical fixation segments (*P* < 0.001), implanted screws (*P* < 0.001), type of scoliosis (*P* < 0.001), correction angle of scoliosis (*P* = 0.073), and total blood volume (*P* = 0.098) were the potential risk factors of HBL. Multiple linear regression analysis showed that operative time (*P* = 0.003) and the number of surgical fixation segments (*P* = 0.014) were risk factors of TBL, while the number of surgical fixation segments (*P* = 0.004) was a risk factor of HBL.

**Conclusions:**

In AIS patients undergoing posterior internal fixation surgery, the operative time and the number of surgical fixation segments are risk factors of TBL, and the number of surgical fixation segments is a risk factor of HBL. Surgeons need to consider these factors when making surgical strategies for AIS patients.

## 1. Introduction

Posterior pedicle screw internal fixation is the main surgical technique for adolescent idiopathic scoliosis (AIS). This surgical technique can greatly improve spine curvature and clinical outcomes, but it usually requires a long surgical incision and multilevel fixation and thus may result in a large amount of blood loss [1, 2]. Massive blood loss may prolong the period of incision healing and recovery, increase the risk of infection, and even cause organ failure [3]. Although intraoperative blood loss and postoperative drainage blood loss can be directly estimated, blood can still be lost in invisible ways, such as surgical interval and hemolysis. This part of blood loss is called hidden blood loss (HBL) [4, 5]. However, HBL is often overlooked by doctors in clinical scenarios. This would result in delayed recovery, anemia, and other adverse symptoms in patients. Therefore, assessment and accurate calculation of blood loss are very important for guiding clinical treatment.

Previous studies have shown that the HBL can account for 26%-60% of the total blood loss (TBL) in various orthopedic surgeries, and minimally invasive surgery can significantly reduce HBL [6–8]. Other studies have found that the average TBL of patients undergoing posterior spinal fusion surgery can reach 1450 mL, and HBL can also reach 600 mL, with HBL accounting for 42% of TBL [9, 10]. However, studies on HBL in AIS patients undergoing surgical treatment are rare.

In this study, we retrospectively analyzed the clinical data of patients with idiopathic scoliosis who received posterior orthopedic fixation and calculated the TBL and HBL by the formula of Nadler et al. and Gross [4, 5]. By evaluating risk factors of TBL and HBL, this study could provide a theoretical basis for reducing blood loss in AIS surgery.

## 2. Materials and Methods

### 2.1. Patients

The AIS patients who visited the department of spine surgery of the Third Hospital of Hebei Medical University between July 2015 and December 2020 were retrospectively reviewed. The study was approved by the Ethics Committee of the Third Hospital of Hebei Medical University. Informed consent was obtained from each patient or guardian before the study, and all data remained anonymous.

Inclusion criteria:

1. Adolescent patients (10-19 years old) with idiopathic scoliosis were treated with simple posterior internal fixation orthopedic surgery, with only varying degrees of posterior column osteotomy and bone grafting without interbody fusion
2. The patient has complete clinical data

Exclusion criteria:

1. The patients suffered from congenital scoliosis, and the presence of spinal dysplasia such as failure of segmentation, hemivertebra, blocked vertebra, and fused vertebra can be observed by X-ray or CT [11]
2. The patients suffered from scoliosis caused by trauma, inflammation, infection, and tumor
3. The patients underwent anterior and middle column osteotomy and intervertebral fusion
4. The patients had hematological diseases, including congenital or acquired diseases such as hemophilia and thrombocytopenic purpura. Coagulation abnormalities due to other organ diseases, drugs, or malnutrition were also excluded
5. The patients had infection, massive bleeding, and other complications during perioperative period

### 2.2. Surgical Procedure and Follow-Up Treatment

All surgeries were performed by the same chief surgeon. During a surgery, the patient was placed in prone position and intubated under general anesthesia. After disinfection, the skin, subcutaneous tissue, and fascia were cut successively to expose the spinous process, lamina, and intervertebral space of the corresponding segment, and pedicle screws were implanted. Small bleeding spots were stopped by electrocoagulation and suture ligation. Part of the posterior column of the spine were removed, including the spinous process, lamina, and articular process. Then, the connecting rods were installed and rotated to correct the scoliosis, pressurize, or expand properly as required. Finally, the nuts were tightened to fix the connecting rod. X-ray fluoroscopy confirmed the correct position of pedicle screws and rods. The drainage tube was placed, and each layer of tissue and skin was sutured successively. The incision was covered with sterile dressings. The anesthesiologists ensured the stability of the patients' respiratory and circulatory system during the surgeries. In particular, we ensured that the patients' blood pressure was within a reasonable range, and there was no large fluctuation. The appropriate use of muscle relaxants facilitated the smooth progress of a surgery.

After surgery, patients were properly rehydrated to maintain body fluid balance. All patients were treated with heparin anticoagulation until discharge. The drainage tube was removed 2-3 days after the operation according to the drainage condition (drainage volume less than 50 mL per day) of the drainage tube.

### 2.3. Data Collection

Basic information (gender, age, weight, height, hospital stay, operative time, intraoperative blood loss, drainage volume, blood transfusion volume, the number of surgical fixation segments and pedicle screws, correction angle of scoliosis, type of scoliosis, and Risser sign) and laboratory test results (hematocrit (HCT) and platelets (PLT) before and after surgery) were collected. The hospital stay was from the day of surgery to the day of discharge. Body mass index (BMI) is calculated according to the World Health Organization (WHO) standards. BMI = Weight (kg)/Height (m)^2^. Drainage volume was the volume of the drainage tube three days after surgery. The type of scoliosis (Lenke classification) and Rissier sign were determined by X-ray and CT. The correction angle of scoliosis was the change of Cobb angle before and after surgery. If double scoliosis or more existed, the change of Cobb angle of maximum scoliosis was taken.

### 2.4. Calculation of TBL and HBL

Nadler formula was used to calculate the total blood volume (TBV) of the patients, and HCT was put into Gross formula to calculate the TBL and HBL of the patients [4, 5].

(1) $$\begin{array}{c} \text{TBV}:\text{K}1*\text{height}{\left(\text{m}\right)}^{3}+\text{K}2*\text{weight}\left(\text{kg}\right)+\text{K}3\left(\text{male}:\text{K}1=0.3669,\text{K}2=0.03219,\text{K}3=0.6041;\text{Female}:\text{K}1=0.3561,\text{K}2=0.03308,\text{K}3=0.1833\right). \end{array}$$

TBL = TBV∗(HCT_pre_ − HCT_post_)/HCT_ave_.HCT_pre_ is the preoperative HCT, and HCT_post_ is the postoperative HCT. The HCT_ave_ is the average of HCT_pre_ and HCT_post_.

Measurable blood loss (MBL) is the sum of intraoperative blood loss (the volume of liquid in the suction bottles volume of lavage fluid used) and drainage blood loss.

(2) $$\begin{array}{c} \text{HBL}:\text{TBL}+\text{blood transfusion volume}-\text{MBL}. \end{array}$$

Since the patient had no significant perioperative bleeding or electrolyte disturbance, we assumed that the TBV of the patient was constant throughout the hospital stay to facilitate the calculation of blood loss.

If the patient is transfused with allogeneic blood, 1 U of red blood cells equals 200 mL of whole blood volume. Drainage blood loss: 20% of the drainage fluid on the first day, 15% of the drainage fluid on the second day, and 5% of the drainage fluid on the third day were calculated as the blood loss in the drainage fluid [10, 12].

### 2.5. Statistical Analysis

Statistical analysis was performed using SPSS 20 (SPSS Inc., Chicago, IL, USA). The measurement data were expressed as mean ± SD. The potential risk factors were assessed by independent sample *t*-test and univariate analysis, *P* < 0.1 indicated potential risk factors. Potential risk factors were included and used in multiple linear regression analysis to investigate risk factors of TBL and HBL; *P* < 0.05 was considered statistically significant.

## 3. Results

A total number of 114 patients with AIS who underwent simple posterior internal fixation orthopedic surgery were enrolled in this study. The clinical data was shown in Table 1. Potential risk factors of TBL and HBL were assessed by univariate analysis. The potential risk factors were included and used in multiple linear regression analysis to investigate the risk factors of TBL and HBL.

As displayed in Table 2, potential risk factors of TBL included operative time (*P* < 0.001), postoperative PLT (*P* = 0.001), the number of surgical fixation segments (*P* < 0.001), implanted screws (*P* < 0.001), hospital stay (*P* = 0.006), type of scoliosis (*P* < 0.001), and correction angle of scoliosis (*P* = 0.063). Potential risk factors of HBL included operative time (*P* < 0.001), postoperative PLT (*P* = 0.095), the number of surgical fixation segments (*P* < 0.001), implanted screws (*P* < 0.001), type of scoliosis (*P* < 0.001), correction angle of scoliosis (*P* = 0.073), and TBV (*P* = 0.098).

Multiple linear regression analysis showed that the operative time (*P* = 0.003) and the number of surgical fixation segments (*P* = 0.014) were risk factors of TBL, as shown in Table 3. The number of surgical fixation segments (*P* = 0.004) was a risk factor of HBL, as shown in Table 4.

## 4. Discussion

Posterior spinal internal fixation is the most common surgical procedure for adolescent idiopathic scoliosis, which can improve the physiological curvature of the spine and relieve pain, improve patients' cardiopulmonary function, and correct patients' attitude towards life [13]. However, multisegment spinal fixation surgery has put forward higher requirements for surgeons, anesthesiologists, and patients' body functions. Massive blood loss from surgery can not only cause hypotension and hypoxia and even affect organ function. Meanwhile, blood transfusion can also increase the risk of complications, such as the spread of infectious diseases [14, 15]. How to reduce perioperative blood loss and promote faster recovery of patients is a hot research topic in the field. Although many reports have pointed out that the use of procoagulant drugs (such as tranexamic acid) can effectively reduce intraoperative blood loss [16]. However, there are few reports on the risk of surgical blood loss and hidden blood loss in adolescents with AIS [1], especially the HBL, which cannot be measured directly with the naked eye. In this study, the clinical data of 114 adolescent scoliosis patients were analyzed to predict the risk factors of TBL and HBL, providing a theoretical basis for guiding doctors to make blood transfusion plans, reduce the risk of related complications, and shorten the recovery period of patients.

In this study, we assessed patients' TBL and HBL. At the same time, TBL (173.51 ± 40.13 mL) and HBL (48.64 ± 13.10 mL) were calculated for each fixation segment, both lower than in previous studies of posterior spinal fusion in adults [9, 17, 18]. This may be related to the younger age and health status of AIS patients [9, 17, 18]. We also calculated the ratio of HBL to TBL (0.29 ± 0.08), which is similar to previous studies, indicating that HBL is a nonnegligible part of TBL [9, 18].

Similar to previous studies, operative time is a risk factor of blood loss [19]. Prolonged operative time leads to prolonged exposure of muscle, bone, and other tissues and increased blood loss during surgery [19]. However, operative time did not significantly increase the patients' HBL, which means the HBL was not caused directly by surgical procedure. And other studies have shown that more than 4 hours of surgery was associated with an increased the risk of bleeding badly in AIS surgery [1]. Therefore, reducing the operative time is an important measure to reduce the amount of operative bleeding on the premise of ensuring the surgical effect and safety.

The increase of fixed segments means longer surgical incision and more muscle dissection [20]. It has been reported that the risk of massive blood loss increased 4.044 times when the number of surgical fixation segments ≥ 10 [21]. Chiu et al. also pointed out that the speed of blood loss during screw placement was the fastest in spinal orthopedic surgery [22]. Wen et al. proved that the number of fixed segments significantly increased HBL in posterior spinal surgery, and significant HBL may be related to postoperative mortality [19]. Our study was consistent with the results of previous studies. Univariate analysis showed that the number of surgical fixation segments and screw implants increased TBL and HBL. However, multiple linear regression analysis showed that only the number of surgical fixation segments was a risk factor of TBL and HBL. Some studies have shown that there was no significant difference in the orthopedic effect of unilateral, bilateral, or bilateral cross screw placement in three dimensions in the case of reducing the number of screws to ensure the surgical effect [23]. Therefore, surgeons need to balance the effect of surgery with the number of fixed segments and reducing blood loss.

Yu et al. found that large preoperative Cobb angle was related to massive intraoperative blood loss [24]. Large preoperative Cobb angle and correction angle require different degrees of orthopedic osteotomy and more internal fixation segments, correspondingly increasing the operative difficulty and time [24]. In this study, although Cobb correction angle was a potential risk factor of TBL and HBL, multiple linear regression analysis showed that the correction angle was not a risk factor. Feeley et al. found that the preoperative Cobb angle, operative time, and intraoperative blood loss of patients with the scoliosis subtype Lenke A/B were lower than those with the scoliosis subtype Lenke C [25]. We also introduced the scoliosis type of Lenke classification into the study to explore the influence of scoliosis type on TBL and HBL. Univariate analysis showed that TBL and HBL of patients with scoliosis subtype 1 was higher than those with scoliosis subtype 5, but scoliosis type was not a risk factor in multivariate linear regression analysis.

This study has some limitations. First, this is a retrospective study, and the sample size is not large because the incidence of AIS is much lower than the other spine diseases. Moreover, some potential risk factors might have been lost due to the limitation of data collection in a retrospective study. Thus, a prospective study with a larger sample size and more risk factors is warranted in the future. Second, for the calculation of intraoperative blood loss, we only included the volume of blood in the suction bottles. We ignored the volume of blood lost through other ways, such as the blood absorbed by gauze. This led to an underestimation of intraoperative blood loss. Finally, blood loss could persist for a long time, especially HBL [26]. In this study, we only investigated blood loss 2-3 days after surgery and did not follow up for a longer period. Therefore, longer follow-up studies are needed to identify the risk factors of blood loss in AIS patients.

## 5. Conclusions

In AIS patients undergoing posterior internal fixation surgery, the operative time and the number of surgical fixation segments are risk factors of TBL, and the number of surgical fixation segments is a risk factor of HBL. Surgeons need to consider decreasing the operative time and the number of surgical fixation segments when making surgical strategies for AIS patients.

## Figures and Tables

**Table 1:** The clinical data of 114 patients who underwent posterior orthopedic surgery for AIS.

Parameters	Mean	SD
Gender (male/female)	38/76	
Age (year)	14.86	4.84
BMI (kg/m^2^)	18.73	3.45
The number of surgical fixation segments	9.62	2.56
The number of pedicle screws	17.14	4.39
Operative time (min)	311.23	117.63
PLT_pre_ (10^9^/L)	235.06	55.28
PLT_post_ (10^9^/L)	190.59	73.92
TBV (mL)	3429.19	720.19
Blood transfusion volume (mL)	822.46	523.59
TBL (mL)	1655.51	564.10
TBL (mL/level)	173.51	40.13
HBL (mL)	475.09	197.59
HBL (mL/level)	48.64	13.10
HBL/TBL	0.29	0.08
Hospital stay (day)	12.26	1.95
Type of scoliosis (1/5/others)	68/37/9	
Risser sign	3.46	1.06
Correction angle (°)	29.39	12.84

AIS: adolescent idiopathic scoliosis; BMI: body mass index; TBL: total blood loss; HBL: hidden blood loss; TBV: total blood volume; PLT_pre_: preoperative platelets; PLT_post_: postoperative platelets.

**Table 2:** The potential risk factors of TBL and HBL.

Parameters	Patients	MBL		*P*	HBL		*P*
		Mean	SD		Mean	SD	
Gender							
Male	38	1724.75	542.07	0.356	504.78	200.43	0.258
Female	76	1620.88	575.18		460.24	195.80	
Age (year)							
≥14	62	1600.31	537.45	0.256	465.81	210.45	0.586
<14	52	1721.33	592.82		486.16	182.50	
BMI (kg/m^2^)							
≥17.88	57	1733.87	628.72	0.139	478.37	194.67	0.860
<17.88	57	1577.15	484.06		471.80	202.15	
Operative time (min)							
≥300	62	1939.87	549.54	<0.001^∗^	586.50	182.39	<0.001^∗^
<300	52	1316.46	358.23		342.26	116.78	
The number of surgical fixation segments							
≥10	58	2007.51	517.46	<0.001^∗^	614.93	162.55	<0.001^∗^
<10	56	1290.93	332.09		330.24	104.14	
The number of pedicle screws							
≥16.5	57	1972.28	539.72	<0.001^∗^	603.93	176.08	<0.001^∗^
<16.5	57	1338.73	382.78		346.25	118.27	
PLT_pre_ (10^9^/L)							
≥234.65	57	1611.10	535.24	0.403	448.19	177.17	0.147
<234.65	57	1699.92	592.96		501.99	214.29	
PLT_post_ (10^9^/L)							
≥177.15	57	1482.81	486.91	0.001^∗^	444.15	181.11	0.095^∗^
<177.15	57	1828.21	586.78		506.03	209.84	
TBV (mL)							
≥3318	57	1568.05	544.20	0.417	459.99	210.36	0.098^∗^
<3318	57	1742.97	574.78		490.19	184.57	
Hospital stay (day)							
≥12	77	1755.56	591.98	0.006^∗^	493.30	195.22	0.157
<12	37	1447.30	439.36		437.19	199.77	
Risser sign							
≥4	69	1654.46	619.98	0.980	482.15	216.48	0.639
<4	45	1657.12	472.423		464.26	166.26	
Type of scoliosis							
1	68	1669.90	566.87	<0.001^∗^	469.08	183.24	<0.001^∗^
5	37	1472.22	438.07		421.81	186.84	
Others	9	2300.27	561.28		739.52	149.25	
Correction angle (°)							
≥28	59	1750.31	555.71	0.063^∗^	507.12	183.29	0.073^∗^
<28	55	1553.80	560.21		440.73	208.07	

^∗^ indicates a potential risk factor (*P* < 0.1). BMI: body mass index; TBL: total blood loss; HBL: hidden blood loss; TBV: total blood volume; PLT_pre_: preoperative platelets; PLT_post_: postoperative platelets.

**Table 3:** Multiple linear regression analysis of risk factors of TBL.

Parameters	Unstandardized		Standardized beta	*t*	*P*	95% CI	
	B	SE					
Operative time (min)	1.644	0.543	0.343	3.026	0.003^∗^	0.567	2.721
PLT_post_ (10^9^/L)	-0.363	0.493	-0.048	-0.736	0.463	-1.341	0.615
The number of surgical fixation segments	89.647	35.733	0.407	2.509	0.014^∗^	18.802	160.491
The number of pedicle screws	6.437	18.661	0.050	0.345	0.731	-30.559	43.434
Hospital stay	8.231	18.543	0.028	0.444	0.658	-28.531	44.994
Type of scoliosis	17.76	19.889	0.059	0.893	0.374	-21.671	57.191
Correction angle (°)	0.529	2.977	0.012	0.178	0.859	-5.374	6.432

^∗^ indicates a risk factor of TBL (*P* < 0.05). PLT_post_: postoperative platelets.

**Table 4:** Multiple linear regression analysis of risk factors of HBL.

Parameters	Unstandardized		Standardized beta	*t*	*P*	95% CI	
	B	SE					
Operative time (min)	0.241	0.186	0.143	1.296	0.198	-0.128	0.609
PLT_post_ (10^9^/L)	0.245	0.169	0.092	1.448	0.150	-0.090	0.581
The number of surgical fixation segments	36.577	12.246	0.474	2.987	0.004^∗^	12.299	60.856
The number of pedicle screws	10.938	6.415	0.243	1.705	0.091	-1.780	23.655
Type of scoliosis	8.579	6.843	0.081	1.254	0.213	-4.989	22.147
Correction angle (°)	-0.721	1.022	-0.047	-0.706	0.482	-2.747	1.304
TBV (mL)	0.018	0.017	0.067	1.061	0.291	-0.016	0.053

^∗^ indicates a risk factor of HBL (*P* < 0.05). PLT_post_: postoperative platelets.

## Data Availability

The data used to support the findings of this study are available from the corresponding author upon request.

## Abbreviations

AIS: Adolescent idiopathic scoliosis
BMI: Body mass index
TBL: Total blood loss
HBL: Hidden blood loss
MBL: Measurable blood loss
TBV: Total blood volume
HCT: Hematocrit
PLT: Platelets.
